# The use of ultra-dense array CGH analysis for the discovery of micro-copy number alterations and gene fusions in the cancer genome

**DOI:** 10.1186/1755-8794-4-16

**Published:** 2011-01-27

**Authors:** Ewa Przybytkowski, Cristiano Ferrario, Mark Basik

**Affiliations:** 1 Department of Oncology, Lady Davis Institute, Sir Mortimer B. Davis Jewish General Hospital, McGill University, Montreal, Quebec, Canada

## Abstract

**Background:**

Molecular alterations critical to development of cancer include mutations, copy number alterations (amplifications and deletions) as well as genomic rearrangements resulting in gene fusions. Massively parallel next generation sequencing, which enables the discovery of such changes, uses considerable quantities of genomic DNA (> 5 ug), a serious limitation in ever smaller clinical samples. However, a commonly available microarray platforms such as array comparative genomic hybridization (array CGH) allows the characterization of gene copy number at a single gene resolution using much smaller amounts of genomic DNA. In this study we evaluate the sensitivity of ultra-dense array CGH platforms developed by Agilent, especially that of the 1 million probe array (1 M array), and their application when whole genome amplification is required because of limited sample quantities.

**Methods:**

We performed array CGH on whole genome amplified and not amplified genomic DNA from MCF-7 breast cancer cells, using 244 K and 1 M Agilent arrays. The ADM-2 algorithm was used to identify micro-copy number alterations that measured less than 1 Mb in genomic length.

**Results:**

DNA from MCF-7 breast cancer cells was analyzed for micro-copy number alterations, defined as measuring less than 1 Mb in genomic length. The 4-fold extra resolution of the 1 M array platform relative to the less dense 244 K array platform, led to the improved detection of copy number variations (CNVs) and micro-CNAs. The identification of intra-genic breakpoints in areas of DNA copy number gain signaled the possible presence of gene fusion events. However, the ultra-dense platforms, especially the densest 1 M array, detect artifacts inherent to whole genome amplification and should be used only with non-amplified DNA samples.

**Conclusions:**

This is a first report using 1 M array CGH for the discovery of cancer genes and biomarkers. We show the remarkable capacity of this technology to discover CNVs, micro-copy number alterations and even gene fusions. However, these platforms require excellent genomic DNA quality and do not tolerate relatively small imperfections related to the whole genome amplification.

## Background

Recent advances in genomics have dramatically increased our capacity to analyze both normal and cancer cells, revealing a multitude of changes in genomic DNA, such as mutations and copy number alterations (CNAs). One of the most exciting discoveries of the last 5 years has been the discovery of the important role of DNA copy number variations or polymorphisms (CNVs) in determining predisposition to diseases such as autism, HIV infection and glomerulonephritis [1-4]. Moreover, the characterization of molecular alterations specific to cancer has enabled the discovery of novel predictive and prognostic biomarkers, which are becoming an integral part of the development of novel targeted therapeutics in cancer. Molecular alterations critical to cancer therapeutics include CNAs such as gene amplifications and deletions as well as genomic rearrangements resulting in gene fusions. DNA amplifications have been shown to contain important druggable oncogenes, such as the genes encoding for the HER2 and EGF receptors [5,6]. The discovery of chromosomal translocations in solid tumors, such as the one involving the *ALK *gene resulting in a novel oncogenic fusion protein in lung adenocarcinoma, have also led to the development of very promising novel therapies directed against these changes [7,8]. Although massively parallel next generation sequencing enables the discovery of such changes [9], this technology remains expensive, requires extensive bioinformatics support, uses considerable quantities of genomic DNA (> 5 ug), and is not easily accessible. On the other hand, a commonly available microarray platform such as array comparative genomic hybridization (array CGH) allows the characterization of gene copy number at a single gene resolution using as little as 0.5 μg of genomic DNA [10]. Such sensitivity becomes important when one considers that genomics technologies are increasingly being applied to minute tumor samples such as those obtained from biopsies. Moreover, the recent development of the one million (1 M) probe array CGH platform by Agilent offers an ultra-high (2.1 kb) resolution definition of DNA copy number alterations. The potential advantage of such ultra-high resolution is the better delineation of DNA breakpoints at DNA copy number alterations as well as the identification of very small, focal CNAs and CNVs.

However, several challenges are posed by the use of such technologies in ever smaller clinical samples. First, how small are the micro-CNAs that can be reliably detected by ultra-high resolution microarrays? Second, can they reliably detect small CNAs using the minute quantities of DNA (e.g. 10-50 ng) extracted from small biopsy samples? In order to obtain enough DNA from such samples, one usually performs whole genome amplification (WGA) of DNA extracted from these samples [11,12]. Does the amplification process introduce artifacts that can confound the analysis of data generated by such high sensitivity technologies [13,14]? As array CGH is increasingly being performed in clinical "biomarker" studies, it is necessary to have a clear understanding of the limitations of this technology in these contexts. To this end, we performed a study to answer two questions: how much sensitivity is gained by using Agilent's 1 M probe array CGH over the less sensitive 244 K arrays, and, can one safely use whole genome amplification of DNA for these array CGH platforms?

Using DNA from the MCF-7 breast cancer cell line, we found that the 4-fold extra resolution of the 1 M array platform led to the improved detection of CNVs and intra-genic CNAs in the MCF-7 cell line, which were mostly less than 100 Kb in genomic length. Interestingly, DNA breakpoints that signal the presence of genomic rearrangements could be detected and better delineated using the ultra high-resolution platform. However, combining the 1 M Agilent array CGH platform with whole genome amplification of DNA results in the appearance of many artifacts, which, although frequently distinguishable from true CNAs by the naked eye, lead to the calling of many spurious CNAs when a commonly used CNA detection algorithm is used. Thus ultra-high resolution methods of detecting CNAs must be used with great caution when WGA is required for the analysis of samples with limiting quantities of DNA.

## Results and discussion

### The detection of micro-copy number alterations using ultra-high resolution array CGH

**Figure 1:**
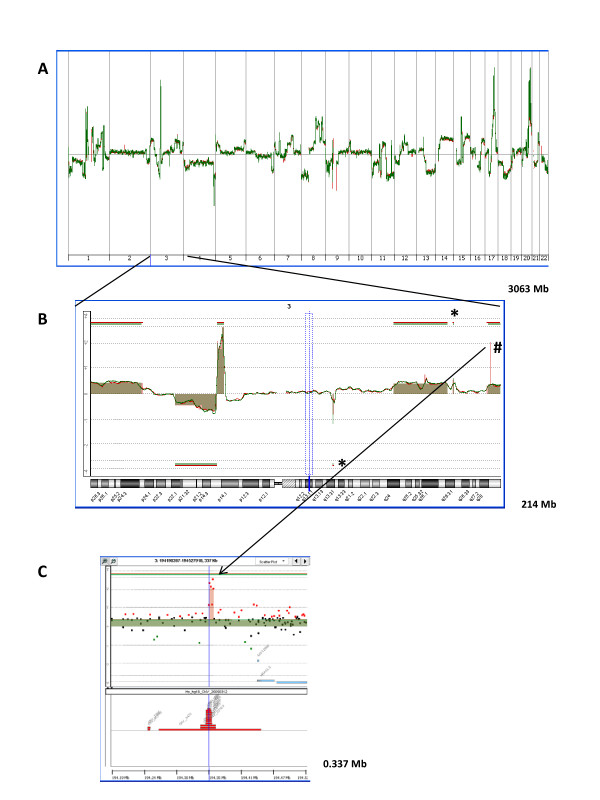
**Copy number alterations (CNAs) found in a genome of MCF-7 cells with ultra-dense array CGH platforms**. Genomic DNA from MCF-7 cells was hybridized to slides containing either 244 K oligonucleotide probes (*green*) or 1 M oligonucleotide probes (*red*). (A) The whole genome view of overlaid moving averages (2 Mb window) for log_2 _ratios of fluorescence between labeled MCF-7 DNA and the differentially labeled normal human reference. (B) Zoom-in on chromosome 3 showing overlaid moving averages and aberrations found with the ADM-2 algorithm. Aberrations smaller than 1 Mb in genomic length are indicated; those found with both 244 K and 1 M platforms (*) and those found with the 1 M platform only (#). (C) Zoom-in on the smallest aberration, 8 Kb amplification, found only with the 1 M platform. Overlaid data points for log_2 _ratios obtained with 1 M platform and 244 K platform are shown (green: values below log_2 _= -0.5; red: values above log_2 _= 0.5; black: values above log_2 _= -0.5 and below log_2 _= 0.5). Aberrations called by the ADM-2 algorithm are identified by a shaded area, and the presence of CNVs is indicated with red boxes (bottom).

To assess the sensitivity of ultra-dense array CGH for the detection of small copy number alterations (CNAs) in the genome we analyzed DNA from MCF-7 cells with 244 K and 1 M array CGH from Agilent. The array CGH data obtained with both platforms were remarkably reproducible at the genomic and chromosomal levels. All large chromosomal aberrations were reliably identified with both platforms (Figure 1A and 1B). We then focused on very small CNAs or "micro-CNAs", defined as those measuring less than 1 Mb in genomic length. To screen for these "micro-CNAs", we used the ADM-2 algorithm developed by Agilent and included in the Agilent Genomic Workbench for CGH analysis. Table 1 lists all 39 such CNAs classified by size, which were found in the MCF-7 genome with the ADM-2 algorithm in both array platforms and includes 24 copy number gains (amplifications) (62%) and 15 copy number losses (deletions) (38%). Three such micro-CNAs found on chromosome 3 are shown in Figure 1B. Two of these contain a single gene, while the third one, which is the smallest CNA detected by the 1 M array CGH, measuring only 8 Kb in genomic length, contains no gene (Figure 1C). In comparison, the smallest CNA found with the 244 K platform measured 64 Kb in genomic length (CNA #8 in Table 1). Thus, the performance of both platforms reflected to some extent the relative spacing of probes on the arrays (i.e. the 4-fold greater resolution of the 1 M arrays).

**Table 1:** All CNAs smaller than 1 Mb, identified with both ultra-dense platforms

#	Chromosome	Start position of CNA found only with 1 M platform	Start position of CNA found with both platforms	Amplification (A) or Deletion (D)	Length of CNA	Presence of known CNV	Number of genes involved	Names of genes involved
1	3	194,358,885		A	8,065	CNV	0	

2	1	150,841,957		D	9,482	CNV	0	

3	7	109,230,336		D	9,874	CNV	0	

4	7	141,698,634		D	15,534	CNV	0	

5	17	4,986,617		A	20,455		1	*USP6*

6	17	59,743,208		A	31,300		1	*PECAM1*

7	1	72,539,143		A	40,168	CNV	0	

8	18		3.210,260	A	64,250		3	*MYOM1* *MYL12A* *MYL12B*

9	6	79,024,557		D	67,293	CNV	0	

10	12	38,617,085		D	68,417		1*	*SLC2A13*

11	12	9,528,590		D	81,464	CNV	0	

12	12	38,437,305		A	91,984		1*	*SLC2A13*

13	15		32,482,458	D	96,025	CNV	1	*GOLGA8A*

14	5		59,959,592	A	133,110		2	*DEPDC1B* *ELOVL7*

15	17		78,519,743	A	133,802		2	*B3GNTL1* *METRNL*

16	20		48,722,374	A	133,933	CNV	2	*BCAS4* *PARD6B*

17	8		39,356,595	A	148,661	CNV	2	*ADAM5P* *ADAM3A*

18	14		37,104,288	A	149,393		1	*FOXA1*

19	20		3,656,779	A	159,562		7	*C20orf27* *MAVS* *C20orf29* *CDC25B* *CENPB* *SPEF1* *HSPA12B*

20	20		55,111,937	A	175,024		1	*BMP7*

21	4	91,703,176		D	178,258		1	*FAM190A*

22	6		151,895,709	A	190,191		2	*C6orf97* *ESR1*

23	1		112,169,367	A	202,264		1	*KCND3*

24	9	21, 842,925		D	221,323	CNV	4	*MTAP* *CDKN2A* *CDKN2B* *CDKN2BAS*

25	20		14,879,882	D	229,686		1	*MACROD2*

26	1		200,010,125	D	242,671		7	*NAV1* *IPO9* *SHISA4* *LMOD1* *TIMM17A* *RNPEP* *ELF3*

27	1		120,065,684	A	349,836	CNV	6	*PHGDH* *HMGCS2* *REG4* *NBPF7* *ADAM30* *NOTCH2*

28	17		56,240,772	A	384,084		1	*BCAS3*

29	7		64,328,811	D	393,567	CNV	3	*ZNF92* *INTS4L1* *INTS4L2*

30	4		182,164,553	D	410,882		0	

31	7		157,963,956	A	413,345		4	*PTPRN2* *NCAPG2* *FAM62B* *WDR60*

32	1	147,203,277		D	441,554	CNV	3	*LOC645166* *LOC388692* *FCGR1C*

33	17		70,788,571	A	541,784	CNV	14	*SLC25A19* *GRB2* *KIAA0195* *CASKIN2* *TSEN54* *LLGL2* *RECQL5* *SAP30BP* *ITGB4* *GALK1* *H3F3B* *UNK* *MYO15B* *LOC643008*

34	9		637,589	A	558,789		4	*KANK1* *DMRT1* *DMRT3* *DMRT2*

35	17		42,299,184	A	602,830		8	*WNT9B* *GOSR2* *RPRML* *CDC27* *MYL4* *ITGB3* *C17orf57* *LOC100272146*

36	3		176,160,173	A	678,671		1	*NAALADL2*

37	3		117,722,695	D	680,165		1	*LOC285194*

38	1	107,928,934		A	910,775		4	*VAV3* *SLC25A24* NBPF4 *NBPF6*

39	12		32, 954,990	A	988,660		1	*SYT10*

Of these 39 micro-CNAs, 15 (38%) were found only using the 1 M platform, and 11 of these were smaller than 100 Kb. Indeed, only 2 of the 13 micro-CNAs smaller than 100 Kb were detected by the 244 K array, while all but 3 of the 26 micro-CNAs greater than 100 Kb in length were detected by both arrays, suggesting that the threshold of sensitivity for the detection of small CNAs for the 244 K array platform is about 100 Kb in chromosomal length. Four CNAs larger than 100 Kb were not detected by the 244 K arrays in our experiments. Two of these were low-level copy number changes, and thus not as likely to be called by the ADM-2 tool, and the other two were better delineated at the higher resolution provided by the 1 M arrays. Of the 15 micro-CNAs detected only by the 1 M array platform, 9 micro-CNAs were localized to sites of common copy number variations (CNVs) as per the Toronto CNV database integrated in the Agilent Genomic Workbench and 7 of these contained no genes (Table 1). Three of these measured less than 10 Kb in genomic length. Since the normal counterpart for MCF-7 cells is not available, it is not possible to determine if these CNVs are truly somatic in this case.

**Figure 2:**
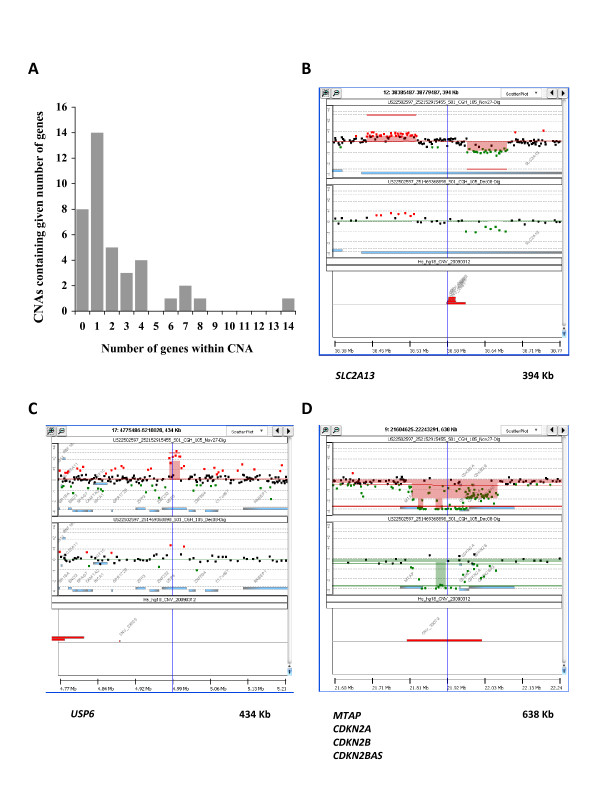
**Micro-CNAs smaller than 1 Mb in genomic length**. (A) Distribution of number of genes within micro-CNAs discovered with both 244 k and 1 M arrays. (B, C and D) Examples of micro-CNAs found only with 1 M platform. Panels show DNA copy number data points and moving averages for the 1 M platform (top, shown in red) or 244 K platform (middle, shown in green). Aberrations are identified by shaded blocks, genes by shaded blue boxes, and the presence of CNVs is indicated with red boxes (bottom). (B) Amplification and deletion within *SLC2A13 *gene. (C) Amplification of *USP6 *gene. (D) Deletion of *MTAP *gene.

Fourteen of the 39 (36%) micro-CNAs involved only a single gene (Figure 2A), including 7 DNA copy gains and 7 DNA copy losses. Five of the 15 micro-CNAs detected only by the 1 M arrays involved one gene each and 3 larger regions involved 3-4 genes each. The five single gene micro-CNAs detected only by 1 M arrays were 3 DNA copy gains and 2 DNA copy losses. One gene (*SLC2A13*) was affected twice, i.e. by a DNA copy gain and a DNA copy loss involving different segments of the gene (Figure 2B), and the 3 other affected genes were: *USP6 *(gain) (Figure 2C), *PECAM1 *(gain), *FAM190A *(loss).

Interestingly, a DNA copy number loss of a small fragment of chromosome 9 next to the *CDKN2A *(p16) gene observed in the 244 K arrays was better mapped in the 1 M arrays, and was found to include the *CDKN2A *(p16) gene as well as the neighboring *MTAP *gene (Figure 2D). The *MTAP *gene has been reported to be a candidate tumor suppressor gene [15,16]. To our knowledge we are the first to report copy number losses of *MTAP *and *CDKN2A *in this cell line and to associate a CNV to this DNA site.

In all, 84 named genes were involved in micro-CNAs (Table 1). We performed a gene ontology search for common biologic processes affected by these genes using the publicly accessible DAVID bioinformatics resources http://david.abcc.ncifcrf.gov, version 6.7. The biologic process category of "cell cycle" was the only gene ontology term enriched with a *p *value < 0.01 in this gene set (*p *= 0.0055). This category included 9 genes: *NOTCH2*, *PARD6B*, *CDKN2A*, *CDKN2B*, *NCAPG2*, *PHGDH*, *CDC27*, *CDC25B*, *LLGL2*. Of note, five genes involved in micro-CNAs were associated with estrogen receptor (ER) signaling: *FOXA1*, *BMP7*, *ESR1 VAV3 *and *PARD6B *[17-20]. Interestingly, *FOXA1 *is a candidate biomarker of poor prognosis in breast tumors [17], *BMP7 *is a biomarker of bone metastasis in breast cancer [21] and *VAV3 *is an oncogene, which maps to a 910Kb amplified region and is known to be overexpressed in MCF-7 cells[19]. Taken together, our findings suggest that ultra-high resolution array CGH, especially the 1 M Agilent platform, leads to the detection of micro-CNAs involving both CNVs and genes with a high degree of sensitivity.

### The detection of breakpoints of chromosomal rearrangements by array CGH

**Figure 3:**
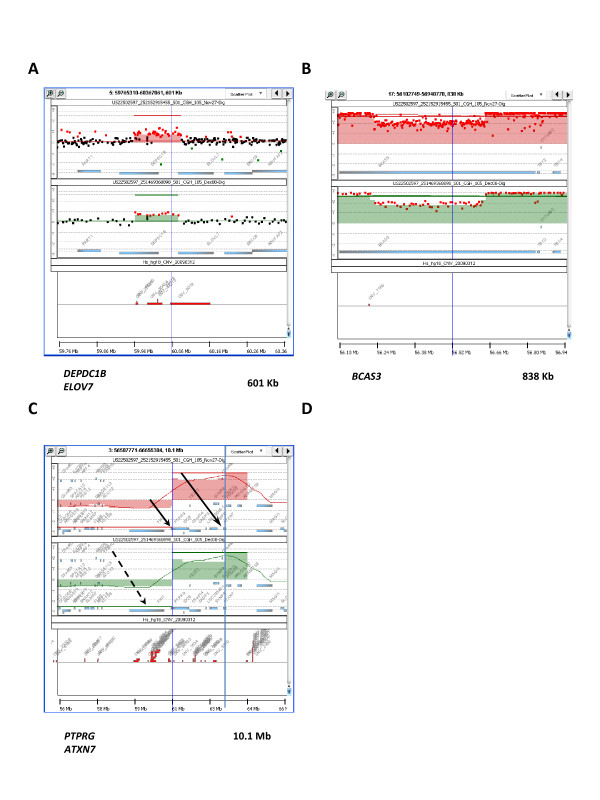
**Three intra-genic "breaks" detected with ultra-dense array CGH analysis, mapping to known gene fusions in the MCF-7 genome**. Each panel shows data points and moving averages for log_2 _ratios of fluorescence between labeled MCF-7 DNA and the differentially labeled normal human reference obtained with 1 M platform (top, shown in red) or 244 K platform (middle, shown in green). Aberrations are identified and the presence of common CNVs is indicated with red boxes (bottom). (A) Amplification affecting *DEPDC1B *and *ELOVL7 *genes. Note that the amplification starts within the *DEPDC1B *gene and ends within the *ELOVL7 *gene, corresponding to an intrachromosomal translocation involving the N-terminus of *DEPDC1B *gene and the C-terminus of the *ELOVL7 *gene [28]. (B) A view of large amplified segment centered around a "relative" DNA copy number loss within the *BCAS3 *(Breast Carcinoma Amplified Sequence 3) gene, corresponding to a gene fusion event involving exons 6-24 or the middle part of the *BCAS3 *gene [28]. (C) Two genes, *PTPRG *and *ATXN7 *(indicated by solid arrows) involved in two different gene fusion events in MCF-7 cells [28] and flanking large amplified segments of chromosome 3 (shaded area) adjacent to the *FRA3B *fragile site, which contains the FHIT gene (broken arrow).

The formation of chromosomal rearrangements such as translocations as well as genomic deletions and amplifications involves double strand DNA breaks [22]. In our data, several genes involved in micro-CNAs (9 genes or 10% of all involved genes) mapped to CNAs in close proximity of known break points or hot spots in chromosomes. Those CNAs were either DNA copy number gains (*USP6*, *NAALADL2*, *BCAS4*, *DEPDC1B*/*ELOVL7*, *BCAS3*) (Figure 2C, 3A and 3B) or losses (*FAM190A*, *MACROD2*, *MTAP*) (Figure 2D) [23-31]. Moreover, using the 1 M array CGH platform, we observed several sites of apparent intra-genic alterations in DNA copy number, suggestive of DNA breakage within genes. We hypothesize that such intra-genic DNA breaks may in some cases indicate gene fusion events. Indeed, recent evidence suggests that such fusion events are more common than previously thought [32]. Hampton et al. recently published a list of gene fusions that involve splicing sites of intact coding exons discovered in the MCF-7 cell line using a parallel sequencing approach [28]. Sixteen distinct genes are involved in these gene fusions in MCF-7 cells, in 4 intra-chromosomal events (1 translocation and 3 inversions) and 6 inter-chromosomal rearrangements, mapping to 6 different chromosomal areas in total (Table 2). Fourteen of these sixteen genes are contained in chromosomal segments affected by DNA copy number gains in the MCF-7 cell line. In our array CGH data, we found that 10 of these 16 genes (Table 2) contained intra-genic copy number alterations, mostly complex changes in DNA copy number. Four of these genes (*DEPDC1B*, *ELOVL7*, *BCAS3 *and *BCAS4*) involved regions of micro-copy number alterations that we identified and listed in Table 1 (Figure 3A and 3B), while the others involved larger chromosomal rearrangements. The one intra-chromosomal translocation involving the *DEPDC1B *and *ELOVL7 *genes was detected as an increase in DNA copy number involving both adjacent genes, but breaking each of them within the gene (Figure 3A). Interestingly, three of the 16 genes (*ARFGEF2*, *SULF2 *and *PRKCBP1*) were contained in one large segment of chromosome 20 affected by DNA copy number gain and two others (*PTPRG *and *ATXN7*) in a large segment of chromosome 3 adjacent to the *FRA3B *fragile site (Figure 3C). Thanks to the ultra-dense spacing of probes on the arrays we were able to break down such large chromosomal segments into smaller regions which differ in copy number values and most likely reflect complex sequence rearrangements (Figure 3B and 3C). These findings suggest that array CGH can also detect chromosomal breaks and rearrangements, which are often accompanied by DNA copy number gains or amplifications. Moreover, ultra-dense array CGH may become a tool to identify gene fusion events similar to what was already suggested for high-resolution single nucleotide polymorphism genomic microarray (SNP-Chip) [33].

**Table 2:** Genes involved in chromosomal rearrangements in MCF-7 cells, as identified by Hampton et al.

Genes	Type of rearrangement	Intra-genic break (detected by array CGH)	Copy number alteration (detected by array CGH)
*ARFGEF2*	intrachromosomal inversion	yes	Amplification

*ASTN2*	interchromosomal	no	

*ATXN7*	interchromosomal	yes	Amplification

*BCAS4*	interchromosomal and intrachromosomal inversion	yes	Amplification

*BCAS3*	interchromosomal	yes	Amplification

*DEPDC1B*	intrachromosomal translocation	yes	Amplification

*ELOVL7*	intrachromosomal translocation	yes	Amplification

*NPEPPS*	intrachromosomal inversion	no	Amplification

*PRICKLE2*	interchromosomal	no	Amplification

*PRKCBP1*	intrachromosomal inversion	no	Amplification

*PTPRG*	interchromosomal	yes	Amplification

*RAD51C*	interchromosomal	no	Amplification

*RSBN1*	interchromosomal	yes	Amplification

*SULF2*	interchromosomal	yes	Amplification

*TBC1D16*	interchromosomal	no	

*USP32*	intrachromosomal inversion	yes	Amplification

### Ultra-dense array CGH analysis reveals micro-amplifications and micro-deletions, which are artifacts inherent to the whole genome amplification

**Figure 4:**
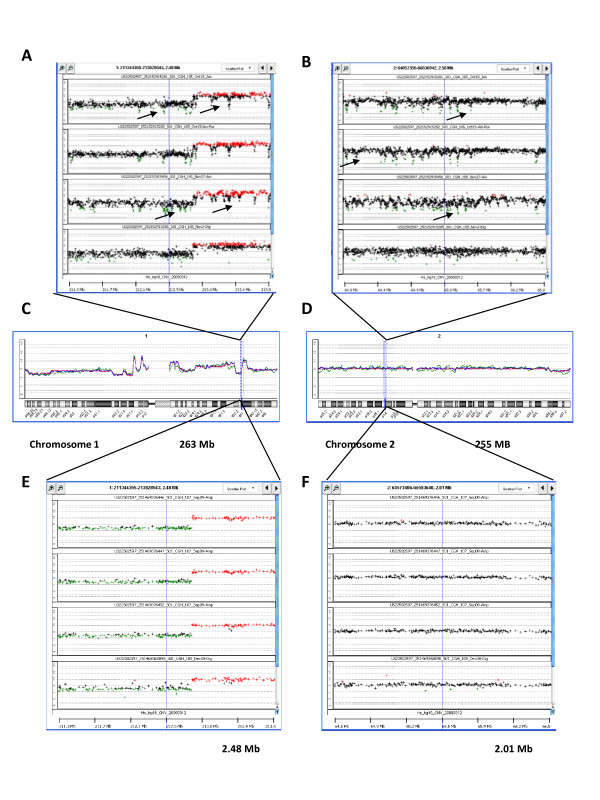
**Array CGH using 1 M platform and whole genome amplified DNA reveals artifacts due to whole genome amplification**. Array CGH of whole-genome amplified DNA from the MCF-7 cell line compared to non-amplified DNA, with magnification of two small segments in chromosomes 1 and 2. (A, B) Data obtained with 1 M platform. In three independent experiments (top 3 sections) DNA was amplified using the Phi29 polymerase kit. The fourth experiment (bottom section) was performed without WGA. Arrows indicate some of the WGA artifacts. (C, D) Array CGH results from the 1 M platform shown as overlaid moving averages obtained in 4 experiments; 3 with WGA-DNA (*blue, green, red*) and 1 without amplification (*purple*). (C) Zoom-out on chromosome 1. (D) Zoom-out on chromosome 2. (E, F) Data obtained with 244 K platform. In three independent experiments (top 3 sections) DNA was amplified using Phi29 polymerase kit. The fourth experiment (bottom section) was performed without WGA. Note the small "wave" effects are seen only in the 1 M arrays when using WGA.

To determine the effect of whole genome amplification (WGA) on the detection of micro-CNAs using the ultra-high density platforms, we compared array CGH results from amplified DNA to non-amplified DNA from the MCF-7 cell line, using both the 1 M and 244 K arrays. The array CGH data obtained with 244 K and 1 M arrays (Figure 4C and 4D) was remarkably reproducible at the genome and chromosomal levels regardless if DNA was amplified or not. However, further magnification to the sub-chromosomal level revealed many repetitive, periodic artifacts in amplified samples (Figure 4A and 4B). This "wave" effect was manifested as the more or less regular periodic appearance of discrete decreases in DNA copy number values spanning about 10-100 Kb, and occurring approximately every 50-500 Kb along each chromosome, with an amplitude of approximately 1-1.5 log_2 _ratio values. These log_2 _ratio value dips were observed in all genomic regions including those of altered copy number (Figure 4A and 4B). This phenomenon considerably confounded the calling of aberrations by the ADM-2 algorithm. We repeated WGA in 3 separate experiments and found that the number of aberrations called by the ADM-2 algorithm in the entire genome varied from 125 in experiment #1 to 561 in experiment #2 and 778 in experiment #3. Since only 39 of those aberrations were found when non-amplified DNA was used for analysis, most of these apparent CNAs are in fact artifacts of DNA amplification. Thus, the number of artifacts greatly exceeded the number of true aberrations. In experiment #1, with the smallest number of artifacts, the majority of them appeared as DNA copy number losses (68.8%). We also found that only 21% of "false" aberrations were found in all three experiments, suggesting that most DNA copy number artifacts are produced randomly during the WGA process. These "wave" artifacts are easily detectable visually in amplified samples analyzed with 1 M platform. Thus, they are not associated specifically with the ADM-2 algorithm. In contrast with the 1 M platform, the use of the 244 K array CGH platform after WGA did not result in such a dramatic number of artifacts. Indeed, the "wave" effect was hardly visible with this platform (Figure 4E and 4F). In three independent experiments performed with amplified DNA the number of aberrations varied from 38 in experiment #1 to 36 in experiment #2 and 35 in experiment #3, compared to a total of 24 micro-CNAs when non-amplified DNA was used for analysis. Thus, the number of potential artifacts was small relative to that found with the denser 1 M platform. In addition, 71% of those artefactual CNAs were common to all three replicates, suggesting that the artifacts observed in this platform may be more dependent on sequence context. Thus, the ultra-dense array CGH platforms, especially the densest 1 M arrays, detect artifacts inherent to WGA and should be used only with non-amplified DNA samples to detect micro-CNAs.

## Conclusion

Our goal is to identify novel targets for therapy and molecular biomarkers with greater precision starting from an in-depth analysis of CNAs present in the breast cancer genome. The advent of ultra-high resolution genomic analysis allows the discovery of novel and very small CNAs hitherto undetectable before, which may involve only single genes. In this first report of the use of the ultra-dense 1 M array CGH Agilent platform for the analysis of DNA from cancer cells, we detected previously unknown intra-genic CNAs affecting genes in the MCF-7 breast cancer cell line, some of which are potentially relevant to cancer biology. Indeed we found that the limit of sensitivity of detection of CNAs of the 244 K array CGH platform is approximately 100 Kb. We have shown that a significant number of smaller micro-CNAs (15 out of total 39, 38%) were only detected by the 1 M array; this includes 9 CNVs as well as two novel amplicons involving the *USP6 *and the *PECAM1 *genes. Micro-CNAs that cut through exonic sequences may indicate potential sites of chromosomal rearrangements and translocations. We found that several gene fusions present in the MCF-7 cell line were also marked by complex intra-genic DNA copy number changes detected by ultra-dense array CGH.

In order to apply these technologies to the kind of small biopsy samples increasingly being collected in modern clinical trials, whole genome amplification is frequently required to obtain sufficient quantities of DNA. Using a commercially available and widely used DNA amplification kit, we found that the higher sensitivity of the 1 M microarray results in the cluttering of the array CGH profile by hundreds of "wave" artifacts. Importantly, these "wave" artifacts do not obscure the detection of true CNAs, even when these are intra-genic and less than 1 Mb in length. On the other hand, the appearance of many artefactual CNAs limits the analysis of the data at the sub-chromosomal level and the use of copy number detection algorithms such as ADM-2. In this study we did not perform a comparison between DNA from fresh or frozen cells versus that extracted from paraffin-embedded samples. In our experience, the genetic material extracted from such samples is of poorer quality and very small focal DNA copy number changes are more difficult to detect. However there is no reason to suppose that the WGA-related artifacts would not be apparent in poorer quality DNA.

Overall, we have demonstrated the remarkable capacity of ultra-dense array CGH platforms for discovery of cancer genes and biomarkers, but we have also shown that such powerful technology requires excellent quality of genomic DNA and does not tolerate relatively small imperfections related to the whole genome amplification.

## Methods

### Cell line

The MCF-7 cell line was cultured in RPMI 1640 (R8758; Sigma, St Louis, MO) supplemented with 10% fetal bovine serum (Hyclone, Logan, UT). Cells in the exponential phase of growth were harvested and DNA extracted using the QuiAmp DNA extraction kit.

### Array CGH

Copy number alterations (CNA) within the MCF-7 genome relative to the sex-matched normal human DNA (Promega, Madison, WI) were identified by array CGH analysis using microarray slides, which contain 244 000 (244 K) and one million (1 × 1 M) oligonucleotide probes (Agilent Technologies, Santa Clara, CA, USA).

For sample preparation and hybridization we have followed the protocol developed and described in detail by Agilent. Briefly, genomic DNA was extracted from MCF-7 cells using QIAmp DNA Mini Kit (Qiagen, Mississauga, Ontario, Canada). The integrity of DNA was confirmed with nanodrop and agarose gel electrophoresis. For array CGH without WGA, we used 2.5 μg of MCF-7 DNA and 2.5 μg of reference DNA for each analysis. DNA was digested with Rsa I and Alu I and labeled by random priming using either Cy5-dUTP or Cy3-dUTP. Following purification with Microcon Centrifugation Filters, Ultracel YM-30 (Millipore, Billerica, Ma, USA), probes were denatured and pre-annealed with 50 μg of human Cot-1 DNA (Invitrogen, Burlington, Ontario, Canada). Hybridization was performed at 65 °C for 40 h with constant rotation.

After hybridization, slides were washed according to the manufacturer's instructions and scanned immediately with a DNA Microarray Scanner (Agilent Technologies). Data were extracted from scanned images using Feature Extraction software, version 10.7.3.1 (Agilent). The text files were then imported for analysis into Genomic Workbench, standard edition 5.0.14 (Agilent). We used the Aberration Detection Method 2 (ADM-2) algorithm to identify DNA copy number aberrations. The ADM-2 algorithm identifies all aberrant intervals in a given sample with consistently high or low log ratios based on the statistical score. It then samples adjacent probes to arrive at an estimation of the true range of the aberrant segment. The statistical score represents the deviation of the average of the log ratios from the expected value of zero, in units of standard deviation. The algorithm searches for intervals in which a statistical score based on the average quality weighted log ratio of the sample and reference channels exceeds a user specified threshold. Although a threshold of 6 is recommended in the instruction manual, we used a conservative threshold of 10 because visual inspection of the array plots led to the rejection of several aberrations called using the lower threshold. We applied a filtering option of minimum of 5 probes in region and minimum absolute average log_2 _ratio > 0.3. USCS human genome assembly hg18 was used as a reference and copy number variations (CNV) were identified with a database integrated in the Agilent Genomic Workbench analytic software.

### Whole genome amplification

For array CGH with WGA, we used 60 ng of both MCF-7 and reference DNA for each analysis. In this case, whole genomic DNA was amplified using GenomiPhi V2 DNA Amplification Kit (GE Healthcare UK Limited, Buckinghamshire, UK), which uses random primers to target the entire DNA template and φ 29 DNA polymerase. WGA generated 7-10 μg of labeled DNA (MCF-7 and reference DNA) for hybridization. Amplified DNA was labeled and purified exactly the same way as digested, non-amplified DNA.

## List of abbreviations

ADM-2: Aberration detection method 2; Array CGH: array comparative genomic hybridization; CNA: copy number alterations; CNV: copy number variations; 1 M array: array CGH containing one million (1 × 1 M) oligonucleotide probes; 244 K array: array CGH containing 244 000 oligonucleotide probes; WGA: whole genome amplification.

## Competing interests

The authors declare that they have no competing interests.

## Authors' contributions

EP: designed the study, carried out all the experiments, performed data analysis, data interpretation and drafted the manuscript; CF: participated in data interpretation and in preparation of the manuscript; MB: conceived the study and participated in its design, data analysis and interpretation and helped to draft the manuscript. All authors read and approved the final manuscript.

## Acknowledgements and funding

We acknowledge support from the Weekend to End Breast Cancer/Jewish General Hospital Foundation, the CIHR funded McGill Integrated Research Training Program (support to E.P.), the National Cancer Institute of Canada (support to C.F.) and the Quebec Breast Cancer Foundation.

## Pre-publication history

The pre-publication history for this paper can be accessed here:

http://www.biomedcentral.com/1755-8794/4/16/prepub
